# AGE-SPECIFIC VIEWS ON INVASIVE AND NON-INVASIVE HUMAN ENHANCEMENTS FOR COGNITIVE DECLINE

**DOI:** 10.1093/geroni/igz038.1193

**Published:** 2019-11-08

**Authors:** G Rainville, Laura Skufca, Madeline Eller

**Affiliations:** AARP, Washington, District of Columbia, United States

## Abstract

This research examines the degree to which younger and older Americans approve of addressing cognitive decline using either a pill-based or an implant-based intervention to restore prior functioning. Half of a probability-based online sample expressed concerns over side effects and levels of approval for a pill-based intervention whereas the remainder of the sample did so for a relatively invasive implant-based enhancement (data were interviews of 2,025 American adults gathered by NORC’s AmeriSpeak panel as part of the AARP Human Enhancements study). We predicted and found that relative disapproval of the implant-based intervention was only significant among those with high concerns over side effects. However, when looking at two age groups for which cognitive decline differed in salience, relative disapproval of the implant-based enhancements were relatively stronger for those 50 and older even among those with few concerns over side effects. This age-based aversion to invasive forms of enhancements may have public health implications in that the subgroup who may most-immediately benefit from the enhancement and may be in the market for only non-invasive enhancements. It is not clear if such enhancements, however, could be delivered via a pill or other non-invasive forms.

